# Extracorporeal Membrane Oxygenation (ECMO) for suspected neonatal genetic diagnoses with cardiorespiratory failure

**DOI:** 10.1051/ject/2023016

**Published:** 2023-09-08

**Authors:** Kelechi Ikeri, Vilmaris Quinones Cardona, Swosti Joshi, Ogechukwu Menkiti

**Affiliations:** 1 Division of Neonatology, Department of Pediatrics, St. Christopher’s Hospital for Children Philadelphia PA 19134 USA; 2 Drexel University College of Medicine Philadelphia PA USA

**Keywords:** Extracorporeal membrane oxygenation (ECMO), Genetic, Pallister-Killian mosaic syndrome, Mitochondrial DNA depletion syndrome, Alveolar capillary dysplasia (ACD)

## Abstract

Recent data describe an increasing use of extracorporeal membrane oxygenation (ECMO) in neonates with various clinical conditions besides primary respiratory or cardiac diagnoses. Infants with underlying genetic disorders characterized by cardiopulmonary failure pose unique management challenges. When pathognomonic dysmorphic features for common genetic diagnoses are not present, the prognosis is uncertain at best when determining ECMO candidacy. Lengthy turnaround times of genetic testing often delay definitive diagnosis during the ECMO course. Clinical management pathways to guide practice and evidence to support the use of ECMO in rare genetic conditions are lacking. The decision to initiate ECMO is daunting but may be of benefit if the subsequent genetic diagnosis is non-lethal. In lethal genetic cases warranting discontinuation of care, the time spent on ECMO may still be advantageous as a bridge to diagnosis while allowing for parental bonding with the terminally ill infant. Diagnostic confirmation may also facilitate the attainment of closure for these parents. Here, we report our experience providing ECMO to three neonates presenting with cardiorespiratory failure and later diagnosed with rare genetic syndromes. We share the challenges faced, lessons learned, and outcomes of these critically ill neonates.

## Overview

Extracorporeal membrane oxygenation (ECMO) is utilized in the management of neonatal respiratory and cardiac failure. Approximately 80% of these neonates have an identifiable primary respiratory diagnosis, while the rest have the primary cardiac disease as the predominant etiology [1]. The use of therapies like inhaled nitric oxide (iNO), exogenous surfactant and high-frequency oscillatory ventilation (HFOV) has resulted in a steady decline in the need for ECMO in neonatal respiratory failure [2]. Over the same period, there has been increasing use of ECMO for neonatal cardiac failure and other clinical scenarios, including genetic conditions [2]. The expanding role of ECMO in this population ushered in an era of increased comfort and willingness to offer ECMO to infants with genetic disorders who have unclear prognoses at the time of decision-making [3].

Lethal genetic syndromes, on the other hand, remain a contraindication to providing ECMO given scarce resources and the futility of therapy [4]. The presence of dysmorphic clinical features not pathognomonic of commonly encountered genetic syndromes may present a diagnostic dilemma. Delays in diagnosis could arise from lengthy turnaround times of genetic testing which could range from 1 to 60 days [5]. Variable expressivity and lack of a definitive diagnosis make an early prediction of neonatal mortality challenging and complicate the process of determining ECMO candidacy. Further compounding these problems is the potential for neonates with underlying but undiagnosed genetic conditions to present with non-specific respiratory and cardiac failure.

Providing ECMO can be beneficial and advantageous in these clinical scenarios where overt contraindications are absent. It affords the team time for a comprehensive diagnostic workup while evaluating the potential for recovery, transplant, or redirection of care when results of expedited genetic testing become available. For parents, obtaining a definitive diagnosis is crucial for attaining closure and understanding its implication for family planning and future generations. For physicians, it facilitates the provision of genetic counseling to families and redirection of care when appropriate.

This report describes three neonatal cases with cardiorespiratory failure diagnosed with rare genetic syndromes after ECMO initiation. The syndromes discussed are Pallister-Killian mosaic syndrome, mitochondrial DNA depletion syndrome, and alveolar capillary dysplasia (ACD). We aim to share experiences providing ECMO in these neonatal cases with suspected genetic diagnoses.

## Description

A summary of all three cases is provided in Tables 1, 2, and 3.

**Table 1:** Patient characteristics.

	Case 1	Case 2	Case 3
Birth weight (g)	3940	2504	2616
Gender	Male	Male	Male
Gestational age, weeks	38.5	38.1	36.1
Diagnosis	HIE, PPHN, COVID-19 exposure	Cardiorespiratory failure with lactic acidosis	PPHN, pneumomedi astinum
Total invasive ventilation, days	22	3	41
Length of stay, days	44	3	41
Survival to discharge	Yes	No	No
Therapy at discharge	None (room air and oral feeds)	NA	NA

HIE, hypoxic ischemic encephalopathy; NA, not available, PPHN, persistent pulmonary hypertension of the newborn.

**Table 2:** ECMO characteristics.

	Case 1	Case 2	Case 3
Pre ECMO			
Arterial blood gas (pH/pCO_2_/pO_2_/HCO_3_/BD)	7.20/41/48/19/-8	7.01/34/255/9/-21	7.17/44/30/16/-12
Oxygenation index	48	44	43
* Inotropic score	35	80	30
*Vasoactive-motropic score	48	40	43
ECMO			
Type	VA	VA	W
Age at initiation (hr of life)	36	23	29
Cannula type	lOFr arterial, 14Fr angled venous	8Fr arterial, lOFr venous	13Fr bicaval dual lumen
Cannulation approach	Central	Peripheral	Peripheral
4-hr post cannulation gas	7.3/40/95/20/-6	7.18/36/246/14/-14	7.23/46/93/19/-9
(pH/pCOi/pOi/HCOj/BD)			
Pump flow range (mL/kg/min)	65–173	50–160	85–154
Sweep flow range (L/min)	0.05–0.24	0.1–0.5	0.1–0.3
Days on ECMO	7	3	14

*Inotropic score (IS) was calculated using dopamine dose (mcg/kg/min) + dobutamine dose (mcg/kg/min) + 100 × epinephrine dose (mcg/kg/min). *Vasoactive-inotropic score (VIS) was calculated using IS + 10 × milrinone dose (mcg/kg/min) + 10,000 × vasopressin dose (units/kg/min) + 100 × norepinephrine dose (mcg/kg/min). BD, base deficit; ECMO, extracorporeal membrane oxygenation; VA, venoarterial; VV, venovenous. pCO_2_ and pO_2_ in mmHg. HCO_3_ in meq/L.

**Table 3:** Genetic features and testing.

	Case 1	Case 2	Case 3
Clinical features	Hypotonia, single simian crease on right hand, micropenis and bifid scrotum.	Family history of neonatal death with unknown cause.	Hypotonia, almond shaped eyes, low set ears, widely spaced nipples, sandal gap deformity.
Biochemical markers		Lactate: 28.7–33.8 mmol/L	
Echocardiographic findings	Large PDA with RVSP 49 + right atrial pressure, moderate tricuspid regurgitation.	Large PDA, severe pulmonary hypertension with severely depressed biventricular function.	Moderate PDA with RVSP 74 + right atrial – pressure, flattened septum, severe RV dilation.
Radiologic testing	Brain MRI: Bilateral ventriculomegaly with interventricular hemorrhage.		Abdominal X-ray suggestive of intestinal obstruction.
	Encephalomalacia and gliosis of cerebral hemisphere.		Chest CTA: Enlargement of main PA. Smaller left lung along with small left.
			PA branches
Suspected diagnosis	Unknown	Electron transport chain deficiency	Alveolar capillary dysplasia
Genetic testing results	FISH: 4 copies ofETV6 and 2 extra copies of short arm of chromosome 12.	WES: 2 heterozygous mutations in the TYMP genes.	WES: de novo heterogeneous Forkhead.
			Box FI (FOXF1) deletion
Day of testing	DOL 2 (prior to ECMO)	DOL 2	DOL 1 and 8
Testing turn-around time	8 days	11 days	14 days

CTA, computed tomography arteriography; DOL, day of life; ECMO, extracorporeal membrane oxygenation; FISH, fluorescence in situ hybridization; MRI, magnetic resonance imaging; PA, pulmonary artery; PDA, patent ductus arteriosus; RV, right ventricle; RVSP, right ventricular systolic pressure; WES, whole exome sequence.

### Case 1

A 3-hour-old 3940 g male infant was transferred to our neonatal intensive care unit (NICU) for neonatal encephalopathy, pulmonary hypertension, and hypoxic respiratory failure. He was born at 38 weeks gestation to a 26-year-old gravida 1 female with a prenatal history significant for maternal COVID-19 infection via emergency cesarean section for recurrent fetal bradycardia and breech presentation. The infant was born limp and apneic requiring positive pressure ventilation. APGAR scores were 2, 6, and 7 at 1, 5 and 10 min of life respectively. He later required intubation and fluid resuscitation. Arterial blood gas showed severe metabolic and lactic acidosis. Examination revealed a hypotonic infant with a single simian crease on the right hand, micropenis, and a bifid scrotum. A brief self-resolved clinical seizure episode was reported. He received intravenous ampicillin and gentamicin for suspected sepsis and was transferred to our NICU.

Therapeutic hypothermia (TH) was started for moderate hypoxic-ischemic encephalopathy (HIE) and respiratory support was changed from conventional to HFOV with the addition of inhaled nitric oxide (iNO) for worsening hypoxemia. An echocardiogram showed a large unrestrictive bi-directional patent ductus arteriosus (PDA), flattened interventricular septum, and supra-systemic right ventricular systolic pressures but normal biventricular function. He was supported with dopamine, dobutamine, vasopressin, and hydrocortisone for hypotension in the setting of persistent pulmonary hypertension of the newborn (PPHN). He developed a left basilar pneumothorax requiring tube thoracocentesis. Fluorescence in situ hybridization (FISH) and microarray testing were sent for suspected genetic diagnosis. With worsening hypoxic respiratory failure and recalcitrant PPHN despite maximal medical therapy, we decided on veno-venous (VV) ECMO support.

A 16Fr Avalon Bi-caval Dual-Lumen cannula (Maquet Getinge Group, Rastatt, Germany) was placed percutaneously in the right internal jugular vein (IJV) by pediatric surgery. This cannula was malpositioned on echocardiography and attempts to reposition or replace it with a 13Fr cannula were unsuccessful. A conversion to veno-arterial (VA) ECMO was achieved by placing a 10Fr arterial cannula (Medtronic, Minneapolis, MN, USA) in the right carotid via cut-down technique followed by a 14Fr angled venous cannula (Medtronic, Minneapolis, MN, USA) centrally placed in the right atrium by cardiothoracic surgery. VA ECMO was initiated at 36 h of life.

A 24-hour video EEG showed no seizures. TH was completed after 72 h. COVID testing done at 24 and 48 h of life was negative. Pressors were weaned, pneumothorax resolved and PPHN improved. The infant was successfully decannulated after 7 days of VA ECMO support.

FISH results reported on DOL 9 showed four copies of ETV6 locus, an isochromosome with two extra copies of the short arm of chromosome 12. Duplication of the terminal region of the short arm of chromosome 12 of ~20Mb was seen on the microarray reported on DOL 16. FISH and microarray results were consistent with Pallister-Killian mosaic syndrome and genetic counseling was provided to the parents. Brain magnetic resonance imaging (MRI) showed bilateral ventriculomegaly, cerebral encephalomalacia and gliosis, and punctate cerebral hemorrhages in the right internal capsule and thalamus consistent with prior insult. He was extubated on DOL 22. He was discharged on DOL 44 in room air and tolerating oral feeds with subspecialty follow-up and developmental surveillance.

### Case 2

A 20-hour-old 2504 g male infant was transferred to our NICU for neonatal encephalopathy, pulmonary hypertension, cardiorespiratory failure, and worsening lactic acidosis. He was born at 38 weeks gestation to a 42-year-old gravida 7 para 5 female via cesarean section for significant fetal bradycardia. Prenatal history was remarkable for intra-uterine growth restriction (IUGR). Family history was significant for a neonatal death at 2 h of life from an unknown cause. His APGAR scores were 8 and 9 at 1 and 5 min of life respectively. He had no discernable dysmorphic features on physical examination. At 14 h of life, he was transferred to the NICU for hypothermia and bradycardia. Blood gas revealed metabolic and respiratory acidosis. Mechanical ventilation and iNO were started for suspected PPHN. He received volume resuscitation for poor perfusion and persistent metabolic acidosis. An echocardiogram showed severely depressed biventricular function, a large PDA with the right to left shunting, supra-systemic right ventricular systolic pressures, moderate mitral valve insufficiency, and a dilated right atrium. Intravenous fluids and epinephrine infusion were initiated for hemodynamic instability. He was lethargic with evidence of poor perfusion including delayed capillary refill.

At our institution, dopamine, milrinone, and hydrocortisone were started. Given the suspicion of metabolic syndrome, a metabolic workup was initiated. Serum amino acids analysis showed elevated proline, glutamine and alanine. Urine organic acids could not be obtained due to anuria. Persistent lactic acidosis and cardiovascular instability led to the initiation of VA ECMO to allow time for comprehensive testing given a notable family history of unexplained neonatal death.

He was placed on VA ECMO via cut-down technique with a 10Fr venous cannula (Medtronic, Minneapolis, MN, USA) in the right IJV and an 8Fr arterial cannula (Medtronic, Minneapolis, MN, USA) in the right carotid artery at 23 h of life. He remained hypotensive with persistent lactic acidosis despite inotropic support, fluid resuscitation and bicarbonate therapy. Given unremitting lactic acidosis and a family history of unexplained neonatal death, a microarray and a whole exome sequence (WES) were obtained and levocarnitine was administered per the genetics team. He required multiple transfusions of packed red blood cells, platelets and fresh frozen plasma for anemia, thrombocytopenia and coagulopathy. Milrinone was started on ECMO on day 2 for poor cardiac function. Furosemide infusion and albumin were given for anuria. Ampicillin and cefepime were continued for presumed sepsis. Video EEG showed suppression of amplitude and discontinuous tracing which was unreactive to stimulus indicative of severe diffuse cerebral dysfunction. Lactic acidosis worsened and hypotension persisted despite being on an ECMO flow of 160 mL/kg/min. Multi-organ failure with likely irreversible cause of lactic acidosis increased suspicion for a lethal mitochondrial disorder warranting redirection to palliative care. After extensive discussion with the family, ECMO flow was weaned gradually and then discontinued at 84 h of life (ECMO day 2). The infant died within a few minutes after discontinuation of ECMO. Results of WES subsequently revealed two heterozygous mutations in the *TYMP* genes (c.668 T > C inherited from his father and c.856 A > G inherited from his mother) leading to a diagnosis of mitochondrial DNA (mtDNA) depletion syndrome.

### Case 3

A 24-hour-old 2616 g male infant was referred to our NICU for persistent pulmonary hypertension and worsening hypoxemia. He was born at 36 weeks to a 27-year-old gravida 2, para 1 female via cesarean section fetal bradycardia. Prenatal history was remarkable for cholestasis and polyhydramnios. APGAR scores were 5 and 8 at 1 and 5 min respectively. The infant was initially on CPAP but worsening hypoxic respiratory failure necessitated intubation, surfactant therapy, HFOV and iNO. He was dysmorphic with low-set ears, widely spaced nipples, global hypotonia and mild sandal gap deformity. Bilious replogle output raised suspicion for gastrointestinal obstruction.

At our institution, he required inotropic support for refractory hypotension. An echocardiogram showed supra-systemic right ventricular pressures with mildly decreased systolic function. Karyotype and microarray were sent on admission. With continued clinical deterioration despite maximal medical management, a 13Fr Avalon Avalon Bi-caval Dual-Lumen catheter (Maquet Getinge Group, Rastatt, Germany) was percutaneously placed and VV ECMO was initiated at 29 h of life. Pulmonary hypertension with worsening right ventricular pressures and hypoxemia persisted on ECMO support necessitating intravenous sildenafil therapy and alprostadil to maintain ductal patency. Based on minimal clinical improvement, treprostinil was started. Persistent pulmonary hypertension with a lack of improvement in hypoxemia despite ECMO and multiple observed extrapulmonary anomalies increased suspicion for ACD. Computed tomography angiogram (CTA) of the chest revealed asymmetric enlargement of the main pulmonary artery with a relatively smaller caliber of the right and left pulmonary artery branches. Expedited WES was sent on DOL 8 (ECMO day 6) with priority for ACD and pulmonary hypertension panel. Given the high suspicion for ACD, pulmonary vasodilators were introduced during ECMO, with successful decannulation after a 14-day run. Results of expedited whole exome sequencing showed a de novo heterogeneous *Forkhead Box F1* (*FOXF1*) deletion seen in ACD. Medical management with pulmonary vasodilators was continued per parental wishes. However, the infant died at 41 days of life.

## Discussion

Advances in genetics have offered providers more comprehensive options for confirmatory testing. It has deepened the understanding of disease processes and increased the likelihood of definitive diagnosis. These factors have revolutionized the care and management of neonates with genetic conditions. Neonates with genetic disorders once considered futile are now being offered newer medical and surgical therapies. In the 1990s, there was a drastic rise in ECMO use in patients with trisomy 21 possibly arising from increased acceptance of this intervention in this population. Lethal chromosomal disorders are a contraindication of ECMO [4]. However, in a survey by Chapman et al., 10% of respondents stated that they would offer ECMO to patients with trisomy 13 and 18 in some instances [3].

In this report, we reflect on the use of ECMO in three distinct cases of neonatal respiratory failure resistant to medical management with a high index of suspicion for genetic disorders. Determining ECMO candidacy in these infants was challenging, given the timing of cardiopulmonary failure and the need for ECMO support between 23 and 36 h of life. Our team consensus favored ECMO initiation with simultaneous diagnostic testing in all three cases.

ECMO therapy is recommended when there is a high likelihood of mortality despite maximal medical therapy and the etiology is potentially reversible [4]. It is an invasive, resource-intensive therapy with inherent risks and complications. Hence, it is reserved for cases with the greatest likelihood of survival. In neonatal cases however, diagnostic uncertainty early in the clinical course and at the time of evaluation for ECMO candidacy makes it challenging to determine survival probability or recovery potential. Reiterer et al. documented a 28-year ECMO center experience with patients with neonatal respiratory failure. They reported a median time of 24 h (range 6–1728 h) from birth to ECMO initiation [6]. Consistent with these findings are the cases presented in our report in which determination of ECMO candidacy was required between 23 and 36 h of life before diagnostic confirmation. However, a unifying theme was the genetics team’s consultation with expeditious genetic testing while maximizing medical therapy and ECMO support.

In case 1, FISH and microarray testing sent before cannulation aided the diagnosis of Pallister-Killian mosaic syndrome after ECMO completion. This rare, non-lethal sporadic disorder is caused by tetrasomy of chromosome 12p arising from the presence of an isochromosome 12p in addition to two normal copies of chromosome 12p. It is a multisystemic condition with varied clinical presentations including hypotonia, craniofacial, cardiac, pulmonary and genitourinary abnormalities. Children with this syndrome often have intellectual disabilities and seizures [7, 8]. This infant demonstrated clinical recovery on ECMO highlighting the beneficial role of ECMO in this case of reversible cardiorespiratory failure with non-lethal genetic diagnosis.

Case 2 describes an infant with severe lactic acidosis recalcitrant to management with an ultimate diagnosis of mtDNA depletion syndrome. Neonatal lactatemia results from electron chain transport dysfunction most commonly secondary to poor tissue oxygenation or a genetic defect [9]. Neonates with hypoxic respiratory failure, cardiac dysfunction, and/or sepsis often present with lactatemia, therefore, we believe it was reasonable to offer ECMO support to maximize oxygen delivery while treating sepsis as a possible source of lactatemia. On the other hand, failure to respond to sufficient ECMO support and other medical interventions in addition to the history of unexplained neonatal death pointed us to a primary lactic acidosis disorder. This guided the decision to re-direct care by ECMO day 2. MtDNA depletion syndrome is a heterogeneous group of inherited metabolic disorders caused by a mutation in 9 genes, one of which is the *TYMP* gene found in this infant. In this disorder, a genetic mutation leads to low levels of mtDNA in specific tissues [10]. Even though mutations in the *TYMP* gene are described in the literature in association with mitochondrial neurogastrointestinal encephalomyopathy (MNGIE) [10], this patient had an unusual presentation with unremitting lactic acidosis characteristic of mitochondrial pathology leading to an ultimately fatal outcome. This case highlights a metabolic condition where ECMO served as a bridge to diagnosis rather than recovery. While ECMO support, in this case, was offered when the diagnosis was unclear, ECMO was discontinued soon as the irreversible nature of the disorder became evident and was not prolonged for genetic confirmation.

Case 3 was diagnosed with ACD which is a rare fatal disorder of lung development often presenting shortly after birth with severe hypoxemia and refractory pulmonary hypertension. Presentation could be atypical with late clinical manifestation and prolonged survival [11]. Extrapulmonary findings are present in 50–80% of cases and commonly observed presentations are the gastrointestinal, genitourinary, and cardiac systems [12, 13]. The FOXF1, FOXC2, and FOXL1 genes on chromosome 16q24.1–q24.2 have been implicated in the pathogenesis of ACD and the FOXF1 gene is seen in 40% of ACD cases [12–14]. Early diagnosis of ACD was made while on ECMO through expedited WES with targeted gene testing for the *FOXF1* gene. Lung biopsy though historically used to diagnose ACD pre-mortem is an invasive procedure typically performed at a median of 6 days on ECMO and leads to a diagnosis in only half of cases [15]. Early lung biopsy allows for prompt diagnosis and discontinuation of therapy in futile cases. An inconclusive or false negative biopsy result is undesirable and problematic. Here, we employed rapid non-invasive genetic testing as a diagnostic alternative based on clinical suspicion and avoided patient exposure to the risks of lung biopsy while on ECMO.

In all three cases, the team decided to initiate ECMO after failed medical management of cardiorespiratory failure. In the first case, ECMO served as a bridge to diagnosis and recovery from hypoxic respiratory failure and PPHN. This infant was discharged home in room air and oral feeds. At the two-year follow-up, this patient had a global developmental delay in receiving early intervention services and continues to have seizures requiring anti-epileptics. In the latter two cases, ECMO served as a bridge to diagnosis. We elected to discontinue ECMO support once there was a strong suspicion of a fatal disorder in the second case, whereas in the third case, our ECMO approach was modified to utilize pulmonary vasodilators to facilitate decannulation and transition to sole medical management.

Non-invasive WES using saliva samples obtained while on ECMO led to a specific diagnosis in two of our patients. In Case 2, ECMO was discontinued before receiving the results of genetic testing when the potential for recovery was deemed unlikely. In Case 3, genetic testing served as a non-invasive diagnostic alternative for the diagnosis of ACD. Previous reports describe the use of similar rapid genetic testing for timely diagnosis of Coffin-Siris syndrome in a 7-month-old infant whose parents subsequently elected for palliative care [16]. Implementation of rapid whole exome and genome sequencing in general has been reported to decrease infant morbidity, resource utilization, and cost of hospitalization [17–19]. Wild et al. found that 63% of surveyed Level IV NICUs were willing to incorporate universal genetic screening of neonates upon cannulation. However, ethical concerns were raised regarding the potential dilemma arising from an increased diagnosis of uncommon genetic conditions where adequate evidence to support or refute the utility of ECMO is lacking [5]. At this time, we suggest an individualized approach and favor early genetic testing when strong clinical suspicion exists, especially in cases with an atypical clinical presentation.

In this case series, the diagnosis secured with WES including inheritance patterns from reported genetic mutations was essential for genetic counseling of parents and determining recurrent risk in subsequent pregnancies. One major psychological stressor for parents is the lack of a specific diagnosis which negatively impacts adaptation and coping [20]. By employing genetic testing while providing ECMO support in these cases valuable information was provided for future family planning. Balancing patient outcomes and family support with resource utilization and healthcare expenditure remains an important aspect of ECMO management that requires further study, especially as it relates to genetic testing.

## Conclusion

This case series highlights the utility of ECMO in critically ill neonates with refractory cardiorespiratory illness and suspected genetic disorders as a bridge to recovery and/or diagnosis. The concomitant use of rapid genetic testing before or during ECMO aids in the timely diagnosis of rare syndromes and impacts the direction of care.

## Data Availability

The research data associated with this article are included in the article.
